# B Cell Composition Is Altered After Kidney Transplantation and Transitional B Cells Correlate With SARS-CoV-2 Vaccination Response

**DOI:** 10.3389/fmed.2022.818882

**Published:** 2022-02-02

**Authors:** Max Schuller, Verena Pfeifer, Alexander H. Kirsch, Konstantin A. Klötzer, Agnes A. Mooslechner, Alexander R. Rosenkranz, Philipp Stiegler, Peter Schemmer, Harald Sourij, Philipp Eller, Barbara Prietl, Kathrin Eller

**Affiliations:** ^1^Division of Nephrology, Department of Internal Medicine, Medical University of Graz, Graz, Austria; ^2^Center for Biomarker Research in Medicine, CBmed GmbH, Graz, Austria; ^3^Division of Endocrinology and Diabetology, Department of Internal Medicine, Medical University of Graz, Graz, Austria; ^4^General, Visceral, and Transplant Surgery, Department of Surgery, Medical University of Graz, Graz, Austria; ^5^Intensive Care Unit, Department of Internal Medicine, Medical University of Graz, Graz, Austria

**Keywords:** kidney transplantation, CKD G5, COVID-19, SARS-CoV-2 vaccination, B cells

## Abstract

**Background:**

The COVID-19 pandemic has major implications on kidney transplant recipients (KTRs) since they show increased mortality due to impaired immune responses to SARS-CoV-2 infection and a reduced efficacy of SARS-CoV-2 vaccination. Surprisingly, dialysis patients have shown superior seroconversion rates after vaccination compared to KTRs. Therefore, we investigated peripheral blood B cell (BC) composition before and after kidney transplantation (KT) and aimed to screen the BC compartment to explain impaired antibody generation.

**Methods:**

A total of 105 patients were recruited, and multicolor flow cytometric phenotyping of peripheral venous blood BC subpopulations was performed before and 1 year after KT. Complete follow-up was available for 71 individuals. Anti-SARS-CoV-2 antibodies were collected retrospectively and were available for 40 subjects, who had received two doses of an mRNA-based vaccine (BNT162b2 or mRNA-1273).

**Results:**

Overall, relative BC frequencies within lymphocytes decreased, and their absolute counts trended in the same direction 1 year after KT as compared to CKD G5 patients. Frequencies and absolute numbers of naïve BCs remained stable. Frequencies of double negative BCs, a heterogeneous subpopulation of antigen experienced BCs lacking CD27 expression, were increased after KT, yet their absolute counts were similar at both time points. Transitional BCs (TrBCs) and plasmablasts were significantly reduced after KT in absolute and relative terms. Memory BCs were affected differently since class-switched and IgM-only subsets decreased after KT, but unswitched and IgD-only memory BCs remained unchanged. CD86^+^ and CD5^+^ expression on BCs was downregulated after KT. Correlational analysis revealed that TrBCs were the only subset to correlate with titer levels after SARS-CoV-2 vaccination. Responders showed higher TrBCs, both absolute and relative, than non-responders.

**Conclusion:**

Together, after 1 year, KTRs showed persistent and profound compositional changes within the BC compartment. Low TrBCs, 1 year after KT, may account for the low serological response to SARS-CoV-2 vaccination in KTRs compared to dialysis patients. Our findings need confirmation in further studies as they may guide vaccination strategies.

## Introduction

Kidney transplantation (KT) is the preferred form of kidney replacement therapy due to superior survival and quality of life compared to dialysis (1).

Solid organ transplantation, however, necessitates strong immunosuppression to avoid graft rejection. Immunosuppressive regimens focus on T-cells and inhibition of acute cellular rejection (2), which has greatly improved early graft survival in the last decades (3). B cells (BCs) have long been overlooked or regarded as “bad guys” due to production of—often detrimental—alloantibodies. In recent years, BCs have been recognized to serve various other important antibody-independent functions in the context of transplantation (4, 5). In that manner, BC-depleting induction therapy has led to more acute cellular rejection episodes (6), and distinct BC subpopulations have been linked to improved graft survival (7, 8). These findings underline the importance of understanding BC heterogeneity and dynamics of BC subsets in KT.

Comparability of studies monitoring BCs after transplantation is hampered by the lack of a uniform classification of human BCs and varying degrees of BC subset resolution. The following major subpopulations can be differentiated in blood using surface antibodies (9): Transitional (Tr) BCs are immature precursors from the bone marrow and switch to naïve BCs in the periphery. Naïve (i.e., antigen inexperienced) BCs, the most abundant peripheral BC subset, circulate in the blood in search of their specific antigen. Upon antigen recognition, they may differentiate into plasmablasts (PBs), plasma cells (PCs), or memory BCs (mBCs). PBs are shor*t-*lived and may migrate to the bone marrow to differentiate into long-lived PCs (10). PBs and PCs secrete highly specific antibodies, which, depending on the antigen, may provide protection against a pathogen, or, in the context of KT, mediate graft rejection. mBCs, the humoral backbone of immune memory, surveil the periphery and quickly mount strong humoral responses upon reencountering of their cognate antigen. Surface expression of IgM and IgD allows further identification of mBCs subpopulations, namely IgM-only (IgM^+^, IgD^−^), IgD-only (IgM^−^, IgD^+^), unswitched [IgM^+^, IgD^+^; often regarded as circulating marginal-zone BCs (11)] and switched (IgM^−^, IgD^−^) mBCs. Finally, double negative (DN) BCs, identified by the lack of surface IgD and CD27 expression, are suggested to comprise early activated memory BCs and extrafollicularly activated precursors of PBs (12).

Immunosuppression in transplantation is a balancing act, with rejection on one side and infection on the other side (13, 14). Infectious diseases contribute significantly to death in kidney transplant recipients (KTRs) (15–17). To avoid preventable deaths from infections, vaccination prior to transplantation is advised, as vaccination efficacy may be impaired in the pos*t-*transplantation setting (18–21).

The COVID-19 pandemic has put KTRs at particular risk. First, more severe and fatal SARS-CoV-2 infections are reported in this population, with an estimated mortality of around 20% of hospitalized KTRs with COVID-19 (22). Second, humoral and cellular immunity after mRNA-based SARS-CoV-2 vaccination and natural infection are markedly reduced in KTRs compared to the general population (23–26). Interestingly, dialysis patients display superior serological responses to mRNA-based vaccination compared to KTRs, almost paralleling results in healthy individuals (24, 25). This difference between dialysis patients and KTRs may be in part explained by the immunosuppressive regimen. However, as other vaccinations have yielded humoral responses despite immunosuppression (19), there might be other factors involved.

Our aim was to explore differences in the BC compartment between the CKD G5 (end-stage kidney disease) setting and the pos*t-*transplantation situation. This study adds to the current knowledge by including previously unstudied BC subsets, such as DN BCs and mBC subpopulations, and by depicting profound compositional changes within BCs 1 year after KT. Furthermore, we provide a potential link between pre-vaccination TrBCs and humoral response to SARS-CoV-2 vaccination in KTRs.

## Materials and Methods

### Study Design and Population

One-hundred-and-five CKD G5 patients were recruited prospectively and transplanted at the Department of General, Visceral, and Transplant Surgery at the Medical University of Graz between 2016 and 2020. All patients were (a) above 18 years old, (b) did not receive immunosuppressive therapy at the time of KT, and (c) received a cadaveric organ. Peripheral blood was collected prior to transplantation (T1) and 1 year after transplantation (T2) for flow cytometric BC characterization and analyses after obtaining informed consent according to the Declaration of Helsinki. Only those with complete follow up and intact allograft at T2 were included. Before KT, patients underwent one hemodialysis session to ensure optimal conditions, and peripheral blood for T1 was taken prior to this session.

The study protocol was approved by the Institutional Review Board of the Medical University of Graz, Austria (28-514ex15/16), and the study was registered as #DRKS00026238 in the German Register of Clinical Studies.

### Vaccination Cohort and Anti-SARS-CoV-2 Antibody Testing

We aimed to investigate if BCs and BC subpopulations correlate with humoral response to an mRNA-based SARS-CoV-2 vaccine. Therefore, out of 71 KTRs, we included those who were available for follow-up, had received two doses of mRNA-1273 (Moderna) or BNT162b2 (Pfizer/BioNTech), had a functioning graft at the time of vaccination, and had finished their last visit before vaccination. Loss of graft function was defined as return to dialysis. In case of symptomatic or asymptomatic COVID-19, detected by either positive RT-PCR or positive serology for anti-SARS-CoV2 nucleocapsid antibody, at any time prior, during, or after vaccination, the patient was excluded.

The time of antibody testing was defined as T3. SARS-CoV-2 specific antibodies against spike protein were measured with LIAISON TrimericS IgG Assay (DiaSorin, Saluggia, Italy), Elecsys Anti-SARS-CoV-2 S (Roche, Basel, Switzerland), Alinity I SARS-CoV-2 IgG (Abbott Laboratories, Chicago, IL, USA) or COV2T (Siemens Healthineers, Erlangen, Germany), depending on where the blood sample was drawn. To allow for comparability between the different test platforms, respective units were converted to BAU/mL using conversion factors, as provided by the manufacturers. Individuals were defined as “responders” in case of detectable antibody levels (i.e., above detection limit) or “non-responders” without detectable antibodies.

### PBMC Isolation and Flow Cytometry Analysis

At both visits, peripheral blood mononuclear cells (PBMCs) were isolated from fresh heparinized whole blood samples (BD vacutainer tubes containing lithium heparin, Becton Dickinson, Franklin Lakes, NJ, USA). Whole blood was diluted 1:1 with phosphate-buffered saline (PBS) and layered into a tube prefilled with Lymphoprep density gradient media (Stemcell Technologies, Vancouver, Canada). Density gradient centrifugation was performed (20 min, 800 × g at room temperature), and the PBMC layer was collected and washed with PBS. Viability and cell number were measured by the use of an automated dual fluorescence cell counter (LUNA-FL, Logos Biosystems, Anyang, South Korea) prior to multi-parameter staining of 1 × 10^6^ cells per FACS panel. Additionally, 0.5 × 10^6^ cells served as an unstained control. Surface panel staining was performed using BD Lyse/Fix buffer (Becton Dickinson) according to the manufacturer's instructions. All antibodies were purchased from Becton Dickinson (for details refer to Supplementary Table 2).

Additionally, 50 μl of fresh whole blood was stained with anti-CD45 APC-H7 antibodies (Becton Dickinson), and 123 count eBeads (Thermo Fisher Scientific, Waltham, MA, USA) were added for the analysis of absolute numbers of leucocyte subpopulations. All samples were acquired on a four-laser BD FACS Fortessa SORP instrument (Becton Dickinson), and data were analyzed using the FlowJo software (Becton Dickinson). UltraComp eBeads (Thermo Fisher Scientific) were used for compensation, and FMO controls were applied for appropriate gating of BC subtypes. The gating strategy is depicted in Supplementary Table 3 and Supplementary Figure 1.

### Statistical Analysis

All statistical analyses were performed with Statistical Package for Social Sciences (SPSS v26, SPSS Inc., Chicago, IL, USA) and GraphPad Prism 8.0.1 (GraphPad Software Inc., San Diego, CA, USA). Graphs were drawn using GraphPad Prism 8.0.1. Normality was assessed by Kolmogorov-Smirnov test. Normally distributed data are shown as mean [standard error of the mean (SEM)], non-normal data as median [interquartile range (IQR)], and categorical data as absolute values and relative frequencies (%). Differences between two independent groups were calculated with *t-*tests, Mann-Whitney U-tests, χ^2^-tests, or Fisher's exact tests, as appropriate. Paired groups were compared using dependent *t-*test or Wilcoxon signed-rank test, depending on the tested variables' distributions. Spearman's rank-based correlation coefficient was used to assess correlations. *P-*values below 0.05 were defined as significant. Formal adjustment for multiple testing was not done.

## Results

### Baseline Characteristics of the Study Population

**Figure 1:**
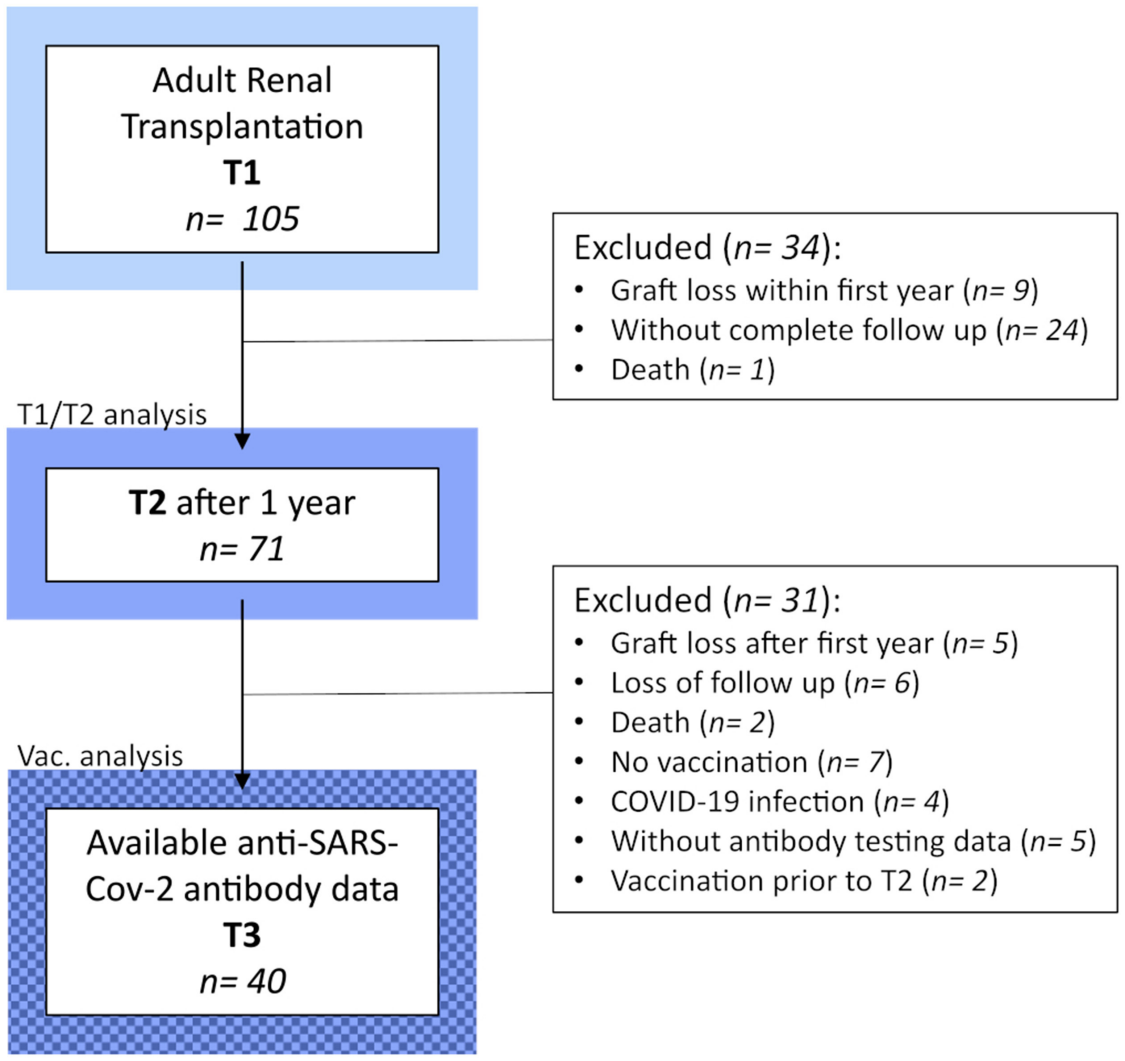
Detailed flow chart of the study design and populations.

Complete follow up, including flow cytometric immune phenotyping at T1 and T2, was available for 71 KTRs (Figure 1). Clinical and demographic characteristics of our T1/T2 cohort are shown in Table 1. The median age was 56 years, and one-third (33.8%) were female. Sixteen patients (22.5%) had diabetes at the time of KT. Participants were mainly Caucasians (94.4%), and only three individuals (4.2%) were transplanted pre-emptively. Of those on dialysis prior to transplantation, hemodialysis was four times more prevalent than peritoneal dialysis (80.9% vs. 19.1%), and median dialysis vintage was 28 months. Glomerular (21.1%) and diabetic kidney disease (18.3%) were the most common causes of CKD G5, followed by polycystic (16.9%) and hypertensive kidney disease (7%). In 36.6% of cases, another cause of CKD was present, or a diagnosis could not be made. Immunosuppressive treatment was prescribed as recommended by the 2009 KDIGO guidelines (18). Briefly, patients with a low pre-transplant risk of rejection received basiliximab (BX; 91.5%), and those with a high risk of rejection were treated with recombinant anti-thymocyte globulin (ATG; 9.9%) as induction therapy. High immunological risk was defined according to the 2009 KDIGO guidelines (18). Of note, pre-transplant panel reactive antibodies (PRAs) over 0% were found in only five individuals One patient's induction therapy had to be switched from ATG to BX due to an allergic reaction. Almost all KTRs were started with triple immunosuppression consisting of corticosteroids (CS; 100%), tacrolimus (TAC; 98.6%), and mycophenolic acid (MPA) or mycophenolate mofetil (MMF) (98.6%), with the exception of one patient, who was started on cyclosporine A (CyA; 1.4%) instead of TAC; and one who received azathioprine (AZA; 1.4%) instead of MMF/MPA. Of note, no KTR received rituximab, and, in those included in the T1/T2 analysis, no rejections were observed within four months of T2.

**Table 1:** Baseline characteristics of study population.

**Variable**	**T1/T2 cohort (*n =* 71)**
Age (years)	56 (48 – 64)
Female sex	24 (33.8)
Body-mass index (kg/m^2^)	26.4 (23.3 – 29.7)
Type 2 diabetes	16 (22.5)
**Ethnicity**	
Caucasian	67 (94.4)
Asian	1 (1.4)
Other	3 (4.2)
**Dialysis prior KT**	68 (95.8)
PD	13 (18.3)
HD	55 (77.5)
Dialysis vintage (months)	28 (20 – 43)
**Renal disease**	
Diabetic	13 (18.3)
Hypertensive	5 (7)
Glomerular	15 (21.1)
Polycystic kidney disease	12 (16.9)
Other/Unknown	26 (36.6)
**Immunosuppression**	
Induction (BX/ATG)	65/7 (91.5/9.9)
TAC	70 (98.6)
CyA	1 (1.4)
MMF/MPA	70 (98.6)
AZA	1 (1.4)
CS	71 (100)
PRAs > 0% prior KT	5 (7)
**Anti-infectives at T2**	
TMP/SMX	0 (0)
Valganciclovir	4 (5.6)
**Rejection within 1st year of KT**	13 (18.3)
Cellular/humoral Rejection	12/1 (92.3/7.7)
BANFF1B	2 (16.7)
BANFF2A	10 (83.3)

*Characteristics of T1/T2 Cohort. Continuous variables are expressed as median (± IQR) and categorical variables as frequency (%). KTR, kidney transplant recipient; KT, kidney transplantation; PD, peritoneal dialysis; HD, hemodialysis; BX, basiliximab; ATG, anti-thymocyte globuline; TAC, tacrolimus; CyA, Cyclosporine A; MMF/MPA, mycophenolate mofetil/mycophenolic acid; AZA, azathioprine; CS, corticosteroids; PRAs, panel-reactive antibodies; TMP/SMX, trimethoprim/sulfamethoxazole*.

### Leucocytes Increase After KT, Whereas Lymphocytes Remain Stable

**Figure 2:**
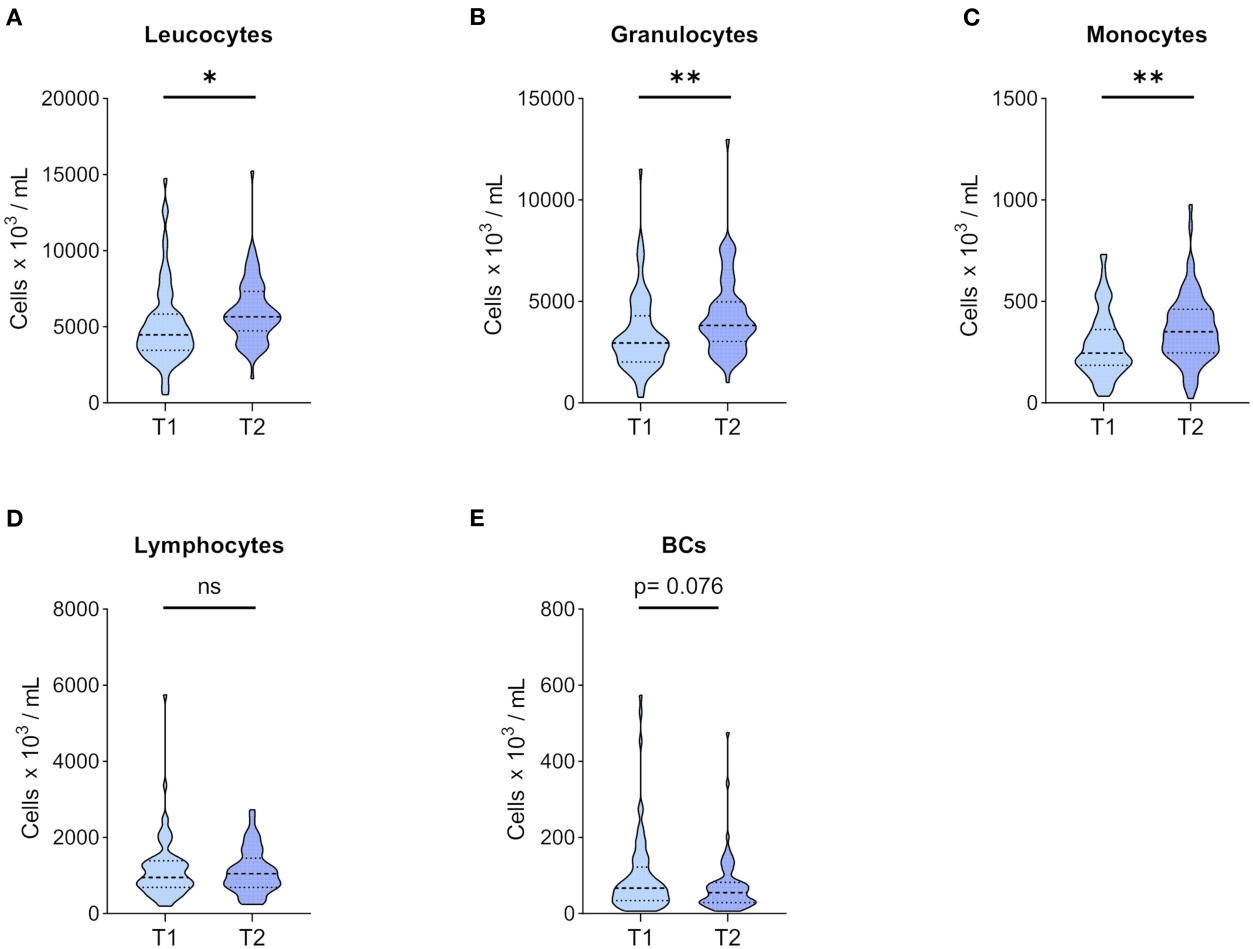
Leucocytes and major leucocyte subpopulation counts. Whole blood of 71 patients was drawn before KT (T1) and 1 year after KT (T2). After staining for CD45, major CD45^+^ leucocyte subpopulations were differentiated according to forward and side scatter using flow cytometry, and absolute numbers were obtained using 123count eBeads (Thermo Fisher Scientific). Violin plots of absolute numbers of **(A)** leucocytes, **(B)** granulocytes, **(C)** monocytes, **(D)** lymphocytes, and **(E)** BCs are shown at T1 and T2. The heavy dashed line indicates the median, and the light dashed lines mark the IQR. The shapes of the colored areas show the data distributions. Differences between both time points were calculated with Wilcoxon signed-rank test (*p < 0.05; **p < 0.01).

Immune cell phenotyping in peripheral blood was performed before KT (T1), which reflects CKD G5 status, and 1 year after KT (T2). A significant increase in leucocytes was observed in patients (Figure 2A) at T2, which was driven by monocyte and granulocyte expansion (Figures 2B,C, respectively). Interestingly, BC numbers showed a marked trend for reduction at T2, missing statistical significance (Figure 2E), whereas total lymphocyte counts remained stable (Figure 2D).

### BC Subpopulations Show Profound Changes 1 Year After KT

**Figure 3:**
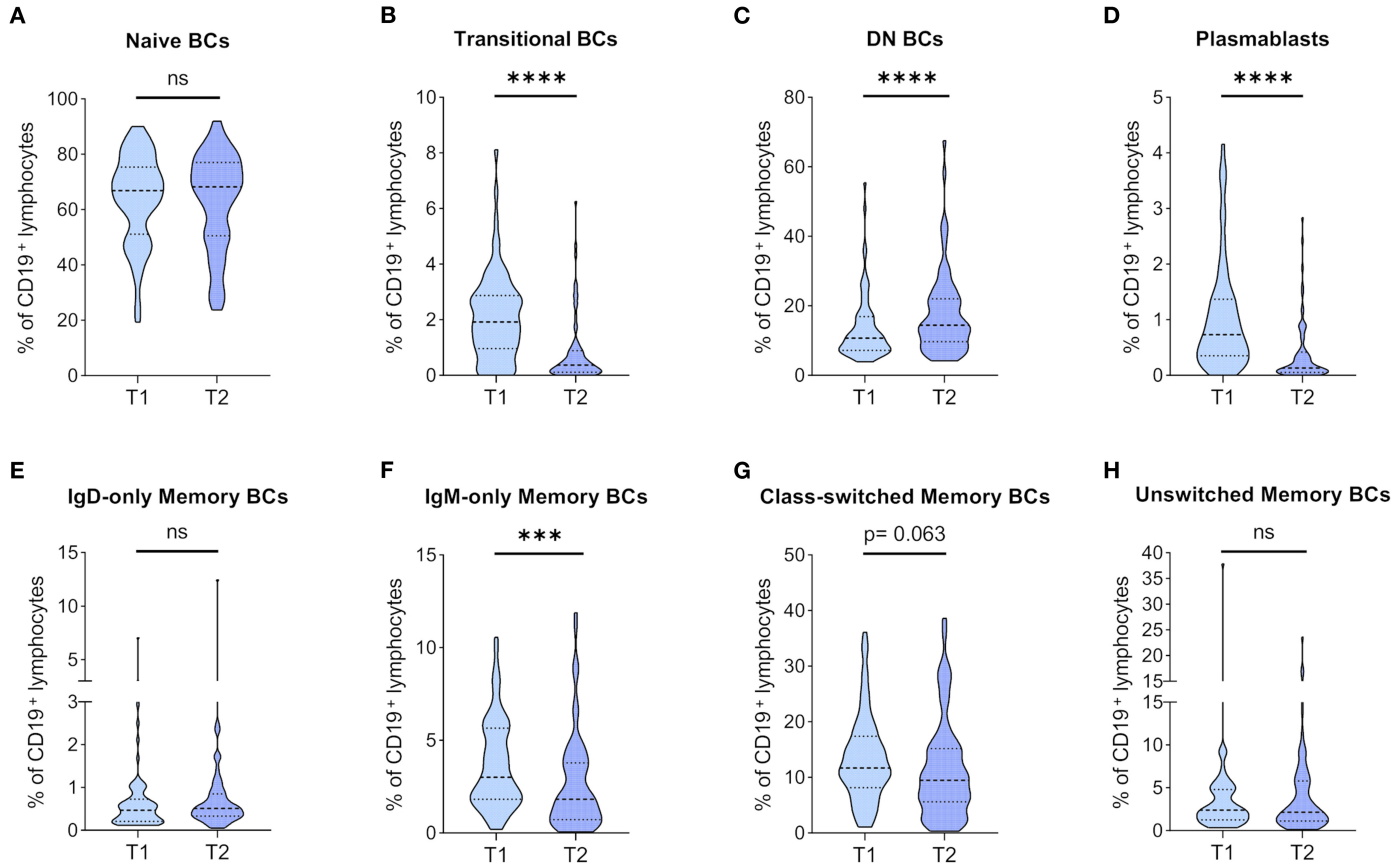
Dynamics of BC subpopulation frequencies before and 1 year after kidney transplantation. PBMCs were analyzed from 71 patients before (T1) and 1 year after KT (T2). Frequencies of **(A)** naïve BCs, **(B)** transitional BCs, **(C)** double-negative (DN) BCs, **(D)** plasmablasts, **(E)** IgD-only memory BCs, **(F)** IgM-only memory BCs, **(G)** class-switched memory BCs, and **(H)** unswitched memory BCs in CD19^+^ lymphocytes are shown as violin plots. Medians are indicated by heavy dashed lines and IQR by light dashed lines, respectively. The distribution of data points is visualized by the plot shape. Wilcoxon signed-rank test was used for comparison between T1 and T2 (***p < 0.001; ****p < 0.0001).

BC subpopulations were monitored, and changes in relative BC frequencies are depicted in Figure 3. Naïve BCs were the major constituent of peripheral BCs, and they were found at similar frequencies at both time points (Figure 3A). TrBCs showed a highly significant reduction in patients 1 year after KT (Figure 3B), whereas DN BCs were significantly upregulated at T2 (Figure 3C). PBs, albeit found at very low frequencies already at T1, were even further reduced after 1 year (Figure 3D). In the mBC compartment, we found significantly decreased IgM-only mBCs in patients 1 year after KT (Figure 3F), whereas class-switched mBCs trended toward lower frequencies at T2 (Figure 3G). Unswitched and IgD-only mBCs remained stable (Figures 3E,H, respectively).

In absolute terms, naïve BCs trended toward a decrease after the first year of KT (Supplementary Figure 2A). TrBC and PB counts were significantly lower at T2 (Supplementary Figures 2B,D, respectively). In contrast to the significant increase of DN BCs with regards to relative frequencies, their absolute numbers were similar at both time points (Supplementary Figure 2C). Quantification of mBC counts revealed findings consistent with relative mBC frequencies. More specifically, pos*t-*transplantation IgM-only mBCs (Supplementary Figure 2F) and class-switched mBCs (Supplementary Figure 2G) were significantly reduced, and IgD-only (Supplementary Figure 2E) and unswitched mBCs (Supplementary Figure 2H) remained stable.

### Activated BCs and Tolerogenic CD27^–^ CD5^+^ BCs Are Decreased After KT

**Figure 4:**
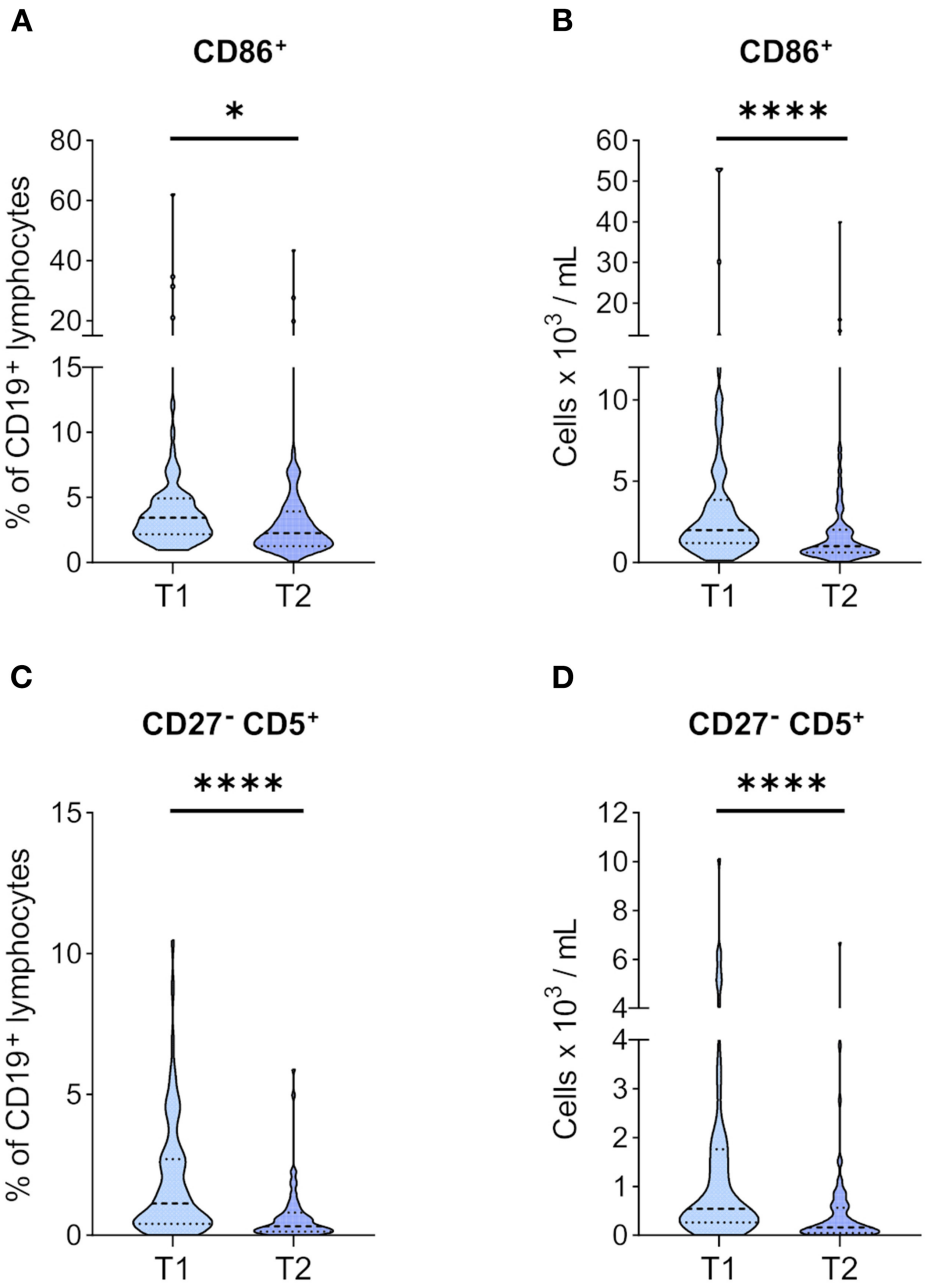
Markers of activation and tolerance in BCs before and 1 year after KT. PBMCs of 71 KTRs before (T1) and 1 year after KT (T2) were stained for CD86, CD27, and CD5. Frequencies of **(A)** CD86^+^ and **(C)** CD27^−^ CD5^+^ in CD19^+^ lymphocytes are given. Absolute numbers per milliliter of **(B)** CD86^+^ and **(D)** CD27^−^ CD5^+^ were calculated from their relative frequency and total BC counts. Median and IQR are marked by heavy and light dashed lines, respectively. The plots shapes indicate the distributions of data points. Comparisons between T1 and T2 were calculated using Wilcoxon-signed rank test (*p < 0.05; ****p < 0.0001).

Next, we were interested in how CD86 expression, an activation marker on BCs, is affected by the immunosuppressive treatment in the post*-*transplantation setting compared to T1. We found CD86^+^ BCs at a significantly lower frequency at T2 than T1 (Figure 4A), which was paralleled by a significant reduction of absolute CD86^+^ BC numbers (Figure 4B). CD5^+^ BCs have gained attention in recent years in the context of transplantation and organ tolerance (27). Therefore, we investigated CD27^−^ CD5^+^ BCs in our cohort and found a significant reduction in their numbers (Figure 4D) and frequencies (Figure 4C).

### MMF Dosage Negatively Impacts on PB Abundance at T2

**Figure 5:**
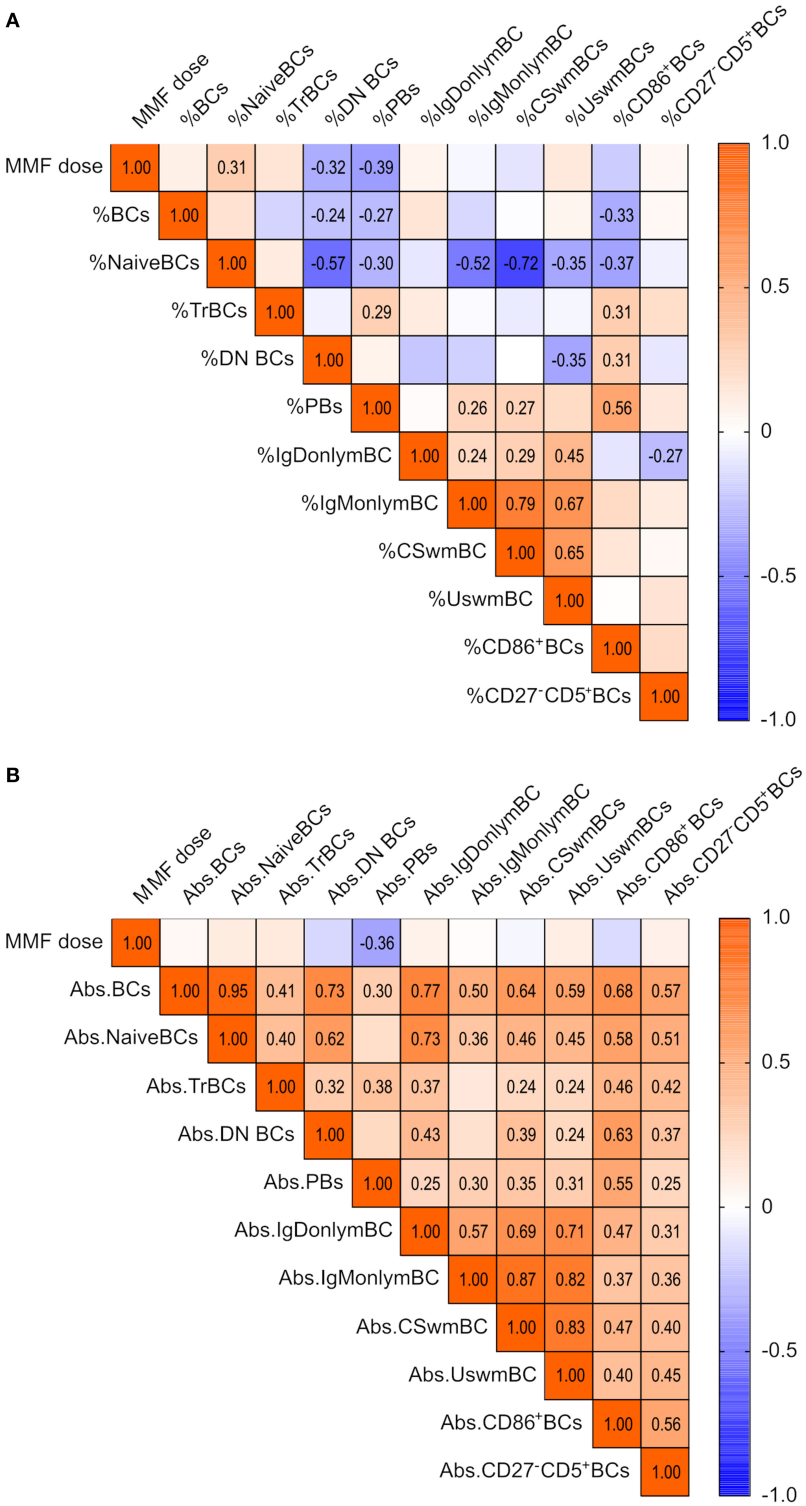
Impact of MMF dose on blood BC pool 1 year after KT in 71 KTRs. Spearman correlation matrix of MMF dose and **(A)** frequencies of BC subpopulations and **(B)** absolute counts of BCs at T2. Correlation coefficients are given for significant correlations only.

Inosine-5′-monophosphate-dehydrogenase is expressed on all lymphocytes, and inhibition via MPA, or its prodrug MMF, targets T and B cells alike (28). MMF was highly prevalent in our cohort (91.5% at T2), yet dosages differed between patients depending on tolerability or infectious complications. Therefore, we investigated, whether MMF dosing affects absolute and relative BCs 1 year after KT. Frequencies of naive BCs positively correlated, and frequencies of DN BCs and PBs negatively correlated with MMF dosage (Figure 5A). In absolute terms, only PBs were negatively correlated with MMF dose (Figure 5B).

### Characteristics of the Vaccination Cohort

To identify potential biomarkers in the BC compartment for a positive serological response following two doses of an mRNA-based vaccine (mRNA-1273 or BNT162b2), administered 4 weeks apart, we retrospectively collected anti-SARS-CoV-2 antibody testing data from our study population. After excluding subjects with previous COVID-19, and those who completed T2 after vaccination, we were able to perform a comparative analysis in 40 patients (Figure 1). Importantly, these individuals displayed very similar dynamics from T1 to T2 in the leucocyte counts and in the BC compartment to the whole study population (Supplementary Figures 3–6). They were also similar with regards to the demographic and clinical characteristics at T1 (Supplementary Table 4).

We identified 20 patients (50%), who mounted a detectable serological response to vaccination (“responders”) and 20 “non-responders” without any detectable anti-SARS-CoV-2 antibodies after two vaccine doses. Anti-SARS-CoV-2 antibody levels were low with a median of 21.75 BAU/mL in responders. Table 2 compares responders to non-responders with regards to their clinical and demographic characteristics at the time of antibody testing (T3).

**Table 2:** Characteristics of vaccination cohort at T3.

**Variable**	**Vaccination subcohort (*n =* 40)**	**Responder (*n =* 20)**	**Non-responder (*n =* 20)**	***p*-value**
Age (years)	64 (54 – 68)^a^	65 (57 – 67)^a^	63 (52 – 68)^a^	0.588
Female Sex	13 (32.5)	6 (30)	7 (35)	1.000
Body-mass index (kg/m^2^)	27.8 (24.8 – 29.6)^b^	27.9 (26.2 – 29.7)^b^	27.6 (24.5 – 30.9)^b^	0.860
Type 2 diabetes	12 (30)	5 (25)	7 (35)	0.731
**Dialysis prior KT**	38 (95)	20 (100)	18 (90)	0.487
PD	9 (23.7)	6 (30)	3 (16.7)	0.451
HD	29 (76.3)	14 (70)	15 (83.3)	1.000
Dialysis vintage (months)	28 (20 – 42.8)	31.5 (21 – 52.75)	26 (15.25 – 40.5)	0.102
**Kidney disease**				
Diabetic	10 (25)	4 (20)	6 (30)	0.716
Hypertensive	3 (7.5)	1 (5)	2 (10)	1.000
Glomerular	7 (17.5)	5 (25)	2 (10)	0.407
Polycystic kidney disease	7 (17.5)	5 (25)	2 (10)	0.407
Other/Unknown	13 (32.5)	5 (25)	8 (40)	0.501
**Rejection**	3 (7.5)	0	3 (15)	0.487
eGFR (mL/min/1.73m^2^)	47.5 (36.6 – 58.7)	49.3 (39.5 – 63.9)	46,8 (36.3 – 54.9)	0.301
Creatinine (mg/dL)	1.44 (1.18 – 1.76)	1.42 (1.11 – 1.78)	1.47 (1.2 – 1.73)	0.602
U-ACR (mg/g)	26.5 (7.3 – 45.5)^c^	28 (14 – 98)^c^	20 (4 – 43)^c^	0.583
U-PCR (mg/g)	107 (77 – 137)^d^	120.5 (78.5 – 161)^d^	93 (77 – 130)^d^	0.298
**Immunosuppression**				
TAC	39 (97.5)	20 (100)	19 (95)	1.000
EVE	3 (7.5)	2 (10)	1 (5)	1.000
MMF/MPA	36 (90)	17 (85)	19 (95)	0.605
<1g/d	17 (47.2)	8 (47.1)	9 (47.4)	1.000
>1g/d	19 (52.8)	9 (52.9)	10 (52.6)	1.000
CS	40 (100)	20 (100)	20 (100)	NA
**Vaccination**				
mRNA-1273/BNT162b2	27/13 (67.5/32.5)	16/4 (80/20)	11/9 (55/45)	0.176
Anti-SARS-CoV-2 antibody level (BAU/mL)	0.59 (0 – 21.78)	21.75 (5.93 – 83.22)	0	<0.001
Interval 2nd dose—T3 (weeks)	15 (9 – 22)^e^	17 (10.3 – 22)^e^	12 (7.5 – 20.8)^e^	0.336
Interval T2 – 1st dose (weeks)	106.5 (85.5 – 151.5)^f^	94 (81 – 148)^f^	132 (85.5 – 159.5)^f^	0.303

*Demographic and clinical characteristics of responders and non-responders to SARS-CoV-2 vaccination. Data are reported as median ± IQR and categorical variables as frequency (%). eGFR, estimated glomerular filtration rate; U-ACR, urinary albumin to creatinine ratio; U-PCR, urinary protein to creatinine ratio; NA, not applicable*.

a *39/19/20 values*;

b *31/14/17 values*;

c *38/19/19 values*;

d *37/18/19 values*;

e *39/19/20 values*;

f *38/20/18 values*.

One-third (*n* = 13) received BNT162b2, and two-thirds (*n* = 27) were given mRNA-1273. In responders, immunization with mRNA-1273 was more frequent than in non-responders (80% vs. 55%). Immunosuppression was very similar to T2, and no significant differences were found in immunosuppressive treatment between the two groups. Importantly, daily MMF/MPA dose was also comparable between responders and non-responders. Furthermore, there were no acute rejections within 6 months prior to T3. The median time difference between T2 and the first dose was 106.5 weeks and not significantly different between both groups. The median time interval from second dose to T3 was 14.5 weeks, and there was also no apparent difference between responders and non-responders. Both groups were at a similar age at the time of T3, and type 2 diabetes was also equally prevalent. There was a trend for longer dialysis vintage prior to T1 in responders (31.5 vs. 26 months), and both preemptively transplanted individuals were found in the non-responder group. Markers of kidney function at T3 did not differ between groups (Table 2).

### TrBCs Correlate With the Anti-SARS-CoV-2 Antibody Response

**Figure 6:**
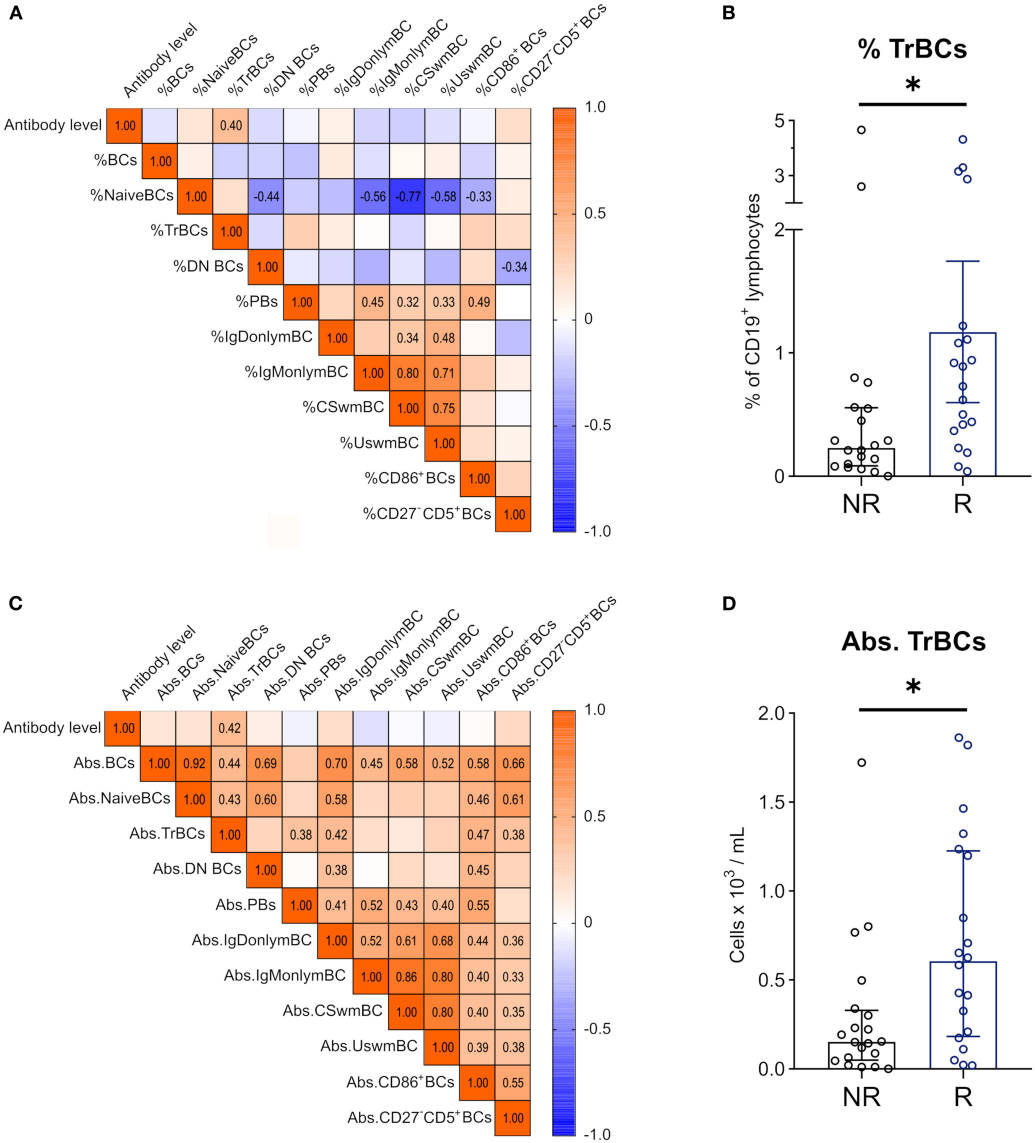
Correlations between anti-SARS-CoV2 antibody levels and BC subpopulations 1 year after KT. Spearman correlation matrix of antibody levels and **(A)** frequencies of BC subpopulations in CD19^+^ lymphocytes 1 year after KT (T2) and **(C)** absolute counts of BC subpopulations at T2. Numbers are given for significant correlations only. Mann-Whitney-U Test was used to compare relative TrBC frequency **(B)** and absolute TrBC numbers **(D)** between non-responders (NR; *n* = 20, black circles) and responders (R; *n* = 20, blue circles). (*p < 0.05).

Correlational analyses of anti-SARS-CoV-2 antibody levels with absolute BC numbers and relative BC frequencies at T2 revealed significant positive correlations of antibodies with relative TrBC frequency of CD19^+^ BCs (Figure 6A) and absolute TrBCs (Figure 6C). Furthermore, when we compared TrBCs in responders to non-responders, we found that TrBC absolute counts, as well as relative TrBC frequency were significantly higher at T2 in those who were able to mount a humoral response to vaccination (Figures 6B,D, respectively).

## Discussion

BCs have gained attention in the field of transplantation in recent years and even more so during the COVID-19 pandemic, as they are at the center of two fundamental problems in solid organ transplantation, namely rejection and infection. Accordingly, numerous studies have monitored BCs after transplantation in search of potential biomarkers for graft rejection (7, 8) and tolerance (29), respectively. On the other hand, few have tried to find a connection between BCs and vaccination success in KT (30), even though the problem of low serological response after transplantation is well known (19) and has become imminent during the COVID-19 pandemic (31).

In this study, we evaluated BCs and BC subpopulations before and 1 year after KT in 71 subjects. These two time points reflect different abnormal immunological steady-states, namely CKD G5 (T1) and stable KT (T2). Accordingly, profound compositional and absolute changes in the BC compartment were detected. Furthermore, we examined whether BCs in stable KTRs are connected to serological response to SARS-CoV-2 vaccination and reported a potential link between pre-vaccination TrBCs and humoral response in KT.

Absolute BCs, detected by CD19 positivity, were not significantly regulated in our hands, which is in line with Svachova et al. (32), who showed that after an initial increase, BCs dropped to pre-transplantation numbers after 1 year, and Schlößer et al. (33), who reported stable levels throughout. Nevertheless, the total BC compartment is reduced compared to healthy individuals (34), supporting the notion that BC numbers are already lower in CKD G5 (35). Immunosuppressive induction and maintenance therapy was very homogenous in our cohort (Table 1). Therefore, we could not assess potential differences in BC composition after KT due to varying immunosuppressive regimen. However, others have shown that after 1 year, KTRs display similar BC pools regardless of ATG or BX induction (32). Furthermore, *in vitro* data suggests that TAC has limited direct effects on BCs (36), but affects humoral immune response in a T-cell mediated manner (37, 38). MMF/MPA, on the contrary, inhibit proliferation of activated lymphocytes, as they depend on rapid *de novo* purine production (28). We observed a dose dependent reduction of PB frequencies and counts by MMF/MPA, but no effect on total BC abundance in correlational analysis. Preferential reduction of PBs as a consequence of MMF/MPA treatment has been reported in SLE as well (39, 40). Additionally, MMF/MPA therapy in KT is a risk factor for failed seroconversion after influenza vaccination (41), and, most recently, SARS-CoV-2 vaccination (24). In summary, these data support the argument that MMF/MPA inhibits antigenic activation and clonal expansion of BCs, but spares non-proliferating subtypes.

Previous studies have shown that there is a compositional switch in KTRs toward a more “differentiated” circulating BC pool (34), and experimental evidence suggests that immature BCs may be more susceptible to immunosuppressive drugs than mature phenotypes (42). In agreement with those findings, we also observed stable numbers of naïve BCs and a significant reduction of TrBCs. However, whereas others have shown stable or increasing mBCs (32, 34), we found reduced IgM-only and switched mBCs and constant levels of IgD-only and unswitched mBCs. These differences may be explained by varying degrees of mBC subset characterization using IgM and IgD. While most describe switched (IgM^−^ IgD^−^) and unswitched (IgM^+^ IgD^+^) mBCs, we report minor subsets, IgM-only (IgM^+^ IgD^−^) and IgD-only (IgM^−^ IgD^+^) mBCs, in KTRs. IgM-only mBCs are widely regarded to originate from germinal centers and to serve as a substrate for switched mBCs (43), but IgD-only mBCs' role and function in health and disease remain largely unknown (44). Evidence hinting toward autoreactivity makes IgD-only mBCs an interesting subset to study in transplantation (45). Thus, our results suggest that not only major BC subpopulations are differently affected by immunosuppression, but also minor subsets.

DN BCs showed an increase in frequency, albeit absolute numbers were similar. DN BCs have been recently recognized as important players in autoimmunity and infectious diseases alike (9, 46), but to our knowledge, their numbers and frequency in blood have not been reported in KTRs. DN BCs may be further characterized by CXCR5 and CD21 expression into functionally distinct subsets, DN1 and DN2, as proposed by Sanz et al. (9). As we did not include these markers in our panel, we were not able to differentiate between DN2, precursors of antibody-secreting cells, which are generated through an extrafollicular pathway; and DN1 BCs, i.e., activated mBCs with a follicular origin (12). DN2 BCs have been shown to be expanded in SLE flares (12), found abundantly in nephritic kidneys (47), and, most recently, have been linked to COVID-19 severity (48, 49). Hence, it will be interesting to monitor these subsets during the course of KT and in the context of rejection and infection specifically.

PBs and PCs may be considered as effector BCs due to their ability to produce antigen-specific antibodies. PCs are mainly located in the bone marrow and are therefore not easily accessible in humans, while PBs can be found in peripheral blood, albeit at low numbers (10). We found PBs to be even further reduced in KTRs, contrasting previous findings, where PBs were shown to repopulate to pre-KT numbers after 1 year (32). Interestingly, others have shown reduced antigen-specific PBs in KTRs and dialysis patients after SARS-CoV-2 vaccination, and post*-*vaccine absolute and relative PBs correlated with antibody levels (50). In our hands, pre-vaccination PBs did not correlate with SARS-CoV-2 antibodies. Taken together, these results suggest impaired generation of new effector BCs, but the size of the already existing PB pool is not predictive of serological response. To clarify this, further data on bone marrow PB and PC kinetics would be of great value.

In contrast to findings from Schlößer et al. (33), who showed that CD86 is downregulated very early after KT, but expression rises back to pre-transplantation levels after 1 year, CD86 was permanently downregulated on BCs in our cohort. Tolerant KTRs, defined as patients with stable kidney function without immunosuppressive medication, have been reported to display higher CD86^+^ BCs compared to non-tolerant KTRs (27, 51). With regards to vaccination, Egli et al. (52), found that pre-vaccination BC expression of CD86 and HLA-DR were correlated with subsequent serological response to H1N1 vaccination. We, however, could not find such a link for SARS-CoV-2 vaccination and CD86^+^ BCs.

In mice, expression of CD5 is used to differentiate between CD5^+^ B1a and CD5^−^ B1b cells, whereas the existence and characterization of human B1 cells are still heavily debated (53–56), and CD5 has been found to be expressed on various human BC subsets (57). CD5 is a negative regulator of BC receptor signaling, and so it is tempting to speculate that higher CD5 expression reduces alloreactive responses in KT (58). Moreover, CD5 promotes the production of IL-10, an inhibitory cytokine, and hallmark for BCs with regulatory functions (Bregs) (59–61). In fact, tolerant KTRs exhibit increased frequencies of blood CD5^+^ BCs compared to stable immunosuppressed KTRs (27), and decreased CD5^+^ BCs were associated with rejection in AB0 incompatible KT (62). We observed a significant reduction in CD27^−^ CD5^+^ BCs at T2. This contrasts previous findings, where CD5^+^ BCs, despite some early fluctuations after transplantation, were found at similar numbers before KT and after the first year (63). Again, these differences may be explained by gating discrepancies. Whereas, we focused on CD5^+^ BCs within the CD27^−^ negative BC population, others have looked at CD5 expression on BCs in general (62, 63). The finding that CD27^+^ mBCs downregulate CD5 supports our gating strategy (64).

In accordance with the existing literature, we have also seen a reduction of TrBCs in the post*-*transplantation setting (32, 65). Low TrBCs have been considered as potential biomarkers for graft rejection (7, 8), and tolerant KTRs display a high frequency of TrBCs (29). Mechanistically, due to their enrichment with Bregs, they may promote tolerance toward the graft (66). Yet, a role of TrBCs in response to vaccination of KTRs has not been described. In our hands, TrBCs significantly correlated with antibody levels after SARS-CoV-2 vaccination in stable KTRs 1 year after transplantation. Moreover, responders to vaccination, characterized as patients with a detectable antibody titer, presented with higher absolute TrBC numbers and higher TrBC frequency in blood than non-responders. In line, Tsang et al. (67) generated a prediction model for serological response to influenza vaccination in healthy individuals, and TrBCs were among the most predictive blood cell populations. However, evidence on pre-vaccination TrBCs in immunocompromised patients is scarce, purely observational, and conflicting. In acute myeloid leukemia patients, a worse response to H1N1 vaccination coincided with higher TrBC frequency than healthy subjects (68). In contrast, TrBCs have also been found abundantly in patients after rituximab therapy, and they responded equally well to H1N1 vaccination as controls with lower TrBCs (69). Nevertheless, patients after rituximab treatment also showed an impaired immune response to SARS-CoV-2 vaccination (70), which might be explained by lower TrBCs, but has not been evaluated so far. Thus, our data warrant further studies to test TrBCs as a potential biomarker for SARS-CoV-2 vaccination success in KTRs and other immunosuppressed patient populations.

The ability to generate specific antibodies to SARS-CoV-2 vaccination indicates intact humoral responsiveness to antigenic stimulation. Accordingly, one may hypothesize that patients with response to SARS-CoV-2 vaccination may also tend to develop allogenic antibodies. Furthermore, it remains to be elucidated whether SARS-CoV-2 vaccination can induce *de novo* formation of donor-specific antibodies (71), as it has been reported for other vaccines (72). Since donor-specific antibodies were not routinely evaluated in our cohort, studies exploring the relationship between allogenic antibodies and SARS-CoV-2 vaccine specific antibodies in transplantation need to be performed in the future.

Our study has several limitations. First, our study was not designed or powered to assess humoral response to SARS-CoV-2 vaccination in a prospective manner. Second, we lack information on peripheral blood leucocytes at the time of vaccination. We cannot rule out changes in the BC compartment from T2 to vaccination, even though immunosuppressive treatment remained similar, and only stable KTRs were assessed. Furthermore, previous reports suggest very little dynamics of BCs several months after KT (34, 73). Third, anti-SARS-CoV-2 antibodies were analyzed on different testing platforms and in different laboratories. Respective values had to be converted to International Standard units for comparison, as recommended by the WHO (74). Though, it is still a matter of debate whether results from different platforms may be used interchangeably (75, 76). We dichotomized the outcome of antibody testing into responders and non-responders, according to the detectability of specific antibodies, with the rationale, that even a minimal response may offer a crude measure of protection. Importantly, there is currently no definitive “protective threshold” for serological response (77).

In conclusion, we have shown a multitude of changes within the BC compartment after KT, including previously not reported subpopulations like DN BCs and mBC subsets. Our results further indicate that pre-vaccination TrBCs in stable KTRs correlate positively with antibody response to SARS-CoV-2 vaccination and are found in higher numbers and frequencies in vaccination responders. Due to low seroconversion rates (23, 24, 78) and emerging evidence of potentially fatal COVID-19 breakthroughs in fully vaccinated transplant recipients (79), it is of major importance to identify those who are unlikely to respond to SARS-CoV-2 vaccination in a timely manner and to emphasize on other means of protection in those individuals. In that manner, the role of TrBCs as pre-vaccination biomarkers for serological response to SARS-CoV-2 and other vaccines in KTRs should be further evaluated.

## Data Availability Statement

The original contributions presented in the study are included in the article/Supplementary Material, further inquiries can be directed to the corresponding author/s.

## Ethics Statement

The studies involving human participants were reviewed and approved by Institutional Review Board of the Medical University of Graz, Austria (28-514ex15/16). The patients/participants provided their written informed consent to participate in this study.

## Author Contributions

MS: acquisition, analysis and interpretation of data, drafting manuscript, and approval of manuscript. VP: acquisition, analysis of data, drafting manuscript, and approval of manuscript. AK: design of study, interpretation of data, reviewing manuscript, and approval of manuscript. KK and AM: interpretation of data, reviewing manuscript, and approval of manuscript. AR, PSc, HS, and PE: design of study, reviewing manuscript, and approval of manuscript. PSt: acquisition of data, reviewing manuscript, and approval of manuscript. BP: design of study, acquisition, analysis and interpretation of data, reviewing manuscript, and approval of manuscript. KE: design of study, acquisition, analysis and interpretation of data, drafting manuscript, and approval of manuscript. All authors contributed to the article and approved the submitted version.

## Funding

This work was supported by the Austrian Science Funds (FWF) to KE. (MOLIN Ph.D. program W1241) and the Austrian National Bank OeNB (Nr.17212 to KE) as well as by an investigator-initiated research grant by Chiesi to KE. MS is a Ph.D. student supported by the MolMed Ph.D. program of the Medical University of Graz.

## Conflict of Interest

Authors VP and BP were employed by CBmed GmbH. The remaining authors declare that the research was conducted in the absence of any commercial or financial relationships that could be construed as a potential conflict of interest.

## Publisher's Note

All claims expressed in this article are solely those of the authors and do not necessarily represent those of their affiliated organizations, or those of the publisher, the editors and the reviewers. Any product that may be evaluated in this article, or claim that may be made by its manufacturer, is not guaranteed or endorsed by the publisher.
