# Supplementary material for: B Cell Composition Is Altered After Kidney Transplantation and Transitional B Cells Correlate With SARS-CoV-2 Vaccination Response
Source: Front Med (Lausanne). 2022 Feb 2;9:818882. doi: 10.3389/fmed.2022.818882 (PMC8847739; doi:10.3389/fmed.2022.818882)
Supplement: Supplementary file 1 [file Data_Sheet_1.PDF]

Supplementary Tables

Supplementary Table 1. List of abbreviations

KT	Kidney transplantation
KTR	Kidney transplant recipient
CKD G5	Chronic kidney disease stage 5 (formerly end-stage kidney disease)
COVID-19	Coronavirus disease 2019
SARS-CoV-2	Severe acute respiratory syndrome coronavirus type 2
PBMCs	Peripheral blood mononuclear cells
BCs	B cells
TrBCs	Transitional B cells
PBs	Plasmablasts
PCs	Plasma cells
mBCs	Memory B cells
DN BCs	Double negative B cells
Bregs	Regulatory B cells
BX	Basiliximab
ATG	Anti-thymocyte globulin
TAC	Tacrolimus
MMF	Mycophenolate mofetil
MPA	Mycophenolic acid
CS	Corticosteroids
EVE	Everolimus
AZA	Azathioprine
CsA	Cyclosporine A
TMP/SMX	Trimethoprim/sulfamethoxazole
PRAs	Panel-reactive antibodies
T1	Timepoint 1 (before transplantation)
T2	Timepoint 2 (one year after transplantation)
T3	Timepoint 3 (anti-SARS-CoV-2 antibody testing)

Supplementary Table 1. List of repeatedly used abbreviations and acronyms.

Supplementary Table 2. Antibodies for B cell characterization.

**Antibody (Clone)**	**Fluorochrome**	**Reference number**
CD19 (SJ25C1)	PE	345789
IgM (G20-127)	BB515	564622
IgD (IA6-2)	PerCP-Cy5.5	561315
CD24 (ML5)	BV711	563401
CD27 (L128)	BV786	563327
CD86 (FUN-1)	PE-CF594	562390
CD38 (HIT2)	APC-R700	564979

Supplementary Table 2. Antibodies used for B cell phenotyping. All antibodies were purchased from Becton Dickinson.

Supplementary Table 3. Gating strategy for B cell subpopulations.

**Reported marker**	**As % of**	**Phenotype**	**Gating: initially gating cells, single cells and lymphocytes**
$CD19^{+}$ B cells in Lymphocytes	Lymphocytes	$CD19^{+}$	(i) Display CD19, on $CD19^{+}$
$IgD^{+}CD27^{-}$ Naive B cells in B cells	$CD19^{+}$ B cells	$CD19^{+}IgD^{+}CD27^{-}$	(i) Display CD19, on $CD19^{+}$, (ii) IgD vs. CD27, on $IgD^{+}CD27^{-}$
Transitional B cells in B cells	$CD19^{+}$ B cells	$CD19^{+}IgD^{+}CD27^{-}$ $CD38^{+}CD24^{+}$	(i) Display CD19, on $CD19^{+}$, (ii) IgD vs. CD27, on $IgD^{+}CD27^{-}$, (iii) CD38 vs. CD24, on $CD38^{+}CD24^{+}$
$IgD^{-}CD27^{-}$ double negative B cells in B cells	$CD19^{+}$ B cells	$CD19^{+}IgD^{-}CD27^{-}$	(i) Display CD19, on $CD19^{+}$, (ii) IgD vs. CD27, on $IgD^{-}CD27^{-}$
$CD38^{+}CD24^{+}$ Plasmablasts in B cells	$CD19^{+}$ B cells	$CD19^{+}IgD^{-/+}CD27^{+}CD38^{+}CD24^{-}$	(i) Display CD19, on $CD19^{+}$, (ii) IgD vs. CD27, combined gate of both $CD27^{+}$ populations, (iii) CD38 vs. CD24, on $CD38^{+}CD24^{-}$
$CD27^{+}CD38^{-/+}CD24^{-/+}$ Memory B cells in B cells	$CD19^{+}$ B cells	$CD19^{+}IgD^{-/+}CD27^{+}CD38^{-/+}CD24^{-/+}$	(i) Display CD19, on $CD19^{+}$, (ii) IgD vs. CD27, combined gate of both $CD27^{+}$ populations, (iii) CD38 vs. CD24, every event except Plasmablasts-Gate
IgD-only Memory B cells in Memory B cells	$CD27^{+}CD38^{-/+}CD24^{-/+}$ Memory B cells in B cells	$CD19^{+}IgD^{-/+}CD27^{+}CD38^{-/+}CD24^{-/+}IgM^{-}IgD^{+}$	(i) Display CD19, on $CD19^{+}$, (ii) IgD vs. CD27, combined gate of both $CD27^{+}$ populations, (iii) CD38 vs. CD24, every event except Plasmablasts-Gate, (iv) IgM vs. IgD, on $IgM^{-}IgD^{+}$
IgM-only Memory B cells in Memory B cells	$CD27^{+}CD38^{-/+}CD24^{-/+}$ Memory B cells in B cells	$CD19^{+}IgD^{-/+}CD27^{+}CD38^{-/+}CD24^{-/+}IgM^{+}IgD^{-}$	(i) Display CD19, on $CD19^{+}$, (ii) IgD vs. CD27, combined gate of both $CD27^{+}$ populations, (iii) CD38 vs. CD24, every event except Plasmablasts-Gate, (iv) IgM vs. IgD, on $IgM^{+}IgD^{-}$
switched Memory B cells in Memory B cells	$CD27^{+}CD38^{-/+}CD24^{-/+}$ Memory B cells in B cells	$CD19^{+}IgD^{-/+}CD27^{+}CD38^{-/+}CD24^{-/+}IgM^{-}IgD^{-}$	(i) Display CD19, on $CD19^{+}$, (ii) IgD vs. CD27, combined gate of both $CD27^{+}$ populations, (iii) CD38 vs. CD24, every event except Plasmablasts-Gate, (iv) IgM vs. IgD, on $IgM^{-}IgD^{-}$
unswitched Memory B cells in Memory B cells	$CD27^{+}CD38^{-/+}CD24^{-/+}$ Memory B cells in B cells	$CD19^{+}IgD^{-/+}CD27^{+}CD38^{-/+}CD24^{-/+}IgM^{+}IgD^{+}$	(i) Display CD19, on $CD19^{+}$, (ii) IgD vs. CD27, combined gate of both $CD27^{+}$ populations, (iii) CD38 vs. CD24, every event except Plasmablasts-Gate, (iv) IgM vs. IgD, on $IgM^{+}IgD^{+}$
$CD86^{+}$ B cells	$CD19^{+}$ B cells	$CD19^{+}CD86^{+}$	(i) Display CD19, on $CD19^{+}$, (ii) CD86 vs. SSC, on $CD86^{+}$
$CD27^{-}CD5^{+}$ B cells in B cells	$CD19^{+}$ B cells	$CD19^{+}CD27^{-}CD5^{+}$	(i) Display CD19, on $CD19^{+}$, (ii) CD5 vs. CD27, on $CD27^{-}CD5^{+}$

Supplementary Table 3. Gating strategy of B cell subpopulations. Frequencies of memory B cell subsets of memory B cells were manually converted to frequencies of memory B cell subsets of B cells for analyses.

Supplementary Table 4. Characteristics of vaccination cohort at T1.

Variable	**Vaccination Cohort (n= 40)**
Age (years)	59.5 (49.5 - 64)
Female sex	13 (32.5)
Body-mass index (kg/m²)	27 (25.2 - 31)
Type 2 diabetes	12 (30)
**Ethnicity**	
Caucasian	37 (92.5)
Asian	1 (2.5)
Other	2 (5)
**Dialysis prior KT**	38 (95)
PD	9 (23.7)
HD	29 (76.3)
Dialysis vintage (months)	28 (20 - 42.8)
**Kidney disease**	
Diabetic	10 (25)
Hypertensive	3 (7.5)
Glomerular	7 (17.5)
Polycystic kidney disease	7 (17.5)
Other/Unknown	13 (32.5)
**Immunosuppression**	
Induction (BX/ATG)	36/5 (90/12.5)
TAC	40 (100)
MMF/MPA	40 (100)
CS	40 (100)
**Rejection within 1st year of KT**	2 (5)
Cellular/humoral Rejection	2/0 (100/0)
BANFF2A	2 (100)

Supplementary Table 4. Clinical and demographic characteristics of Vaccination Cohort at T1. Data are reported as median ± IQR and categorical variables as frequency (%).

KT, kidney transplantation; PD, peritoneal dialysis; HD, hemodialysis; BX, basiliximab; ATG, anti-thymocyte globuline; TAC, tacrolimus; MMF/MPA, mycophenolate mofetil/mycophenolic acid; CS, corticosteroids.

Supplementary Figures:

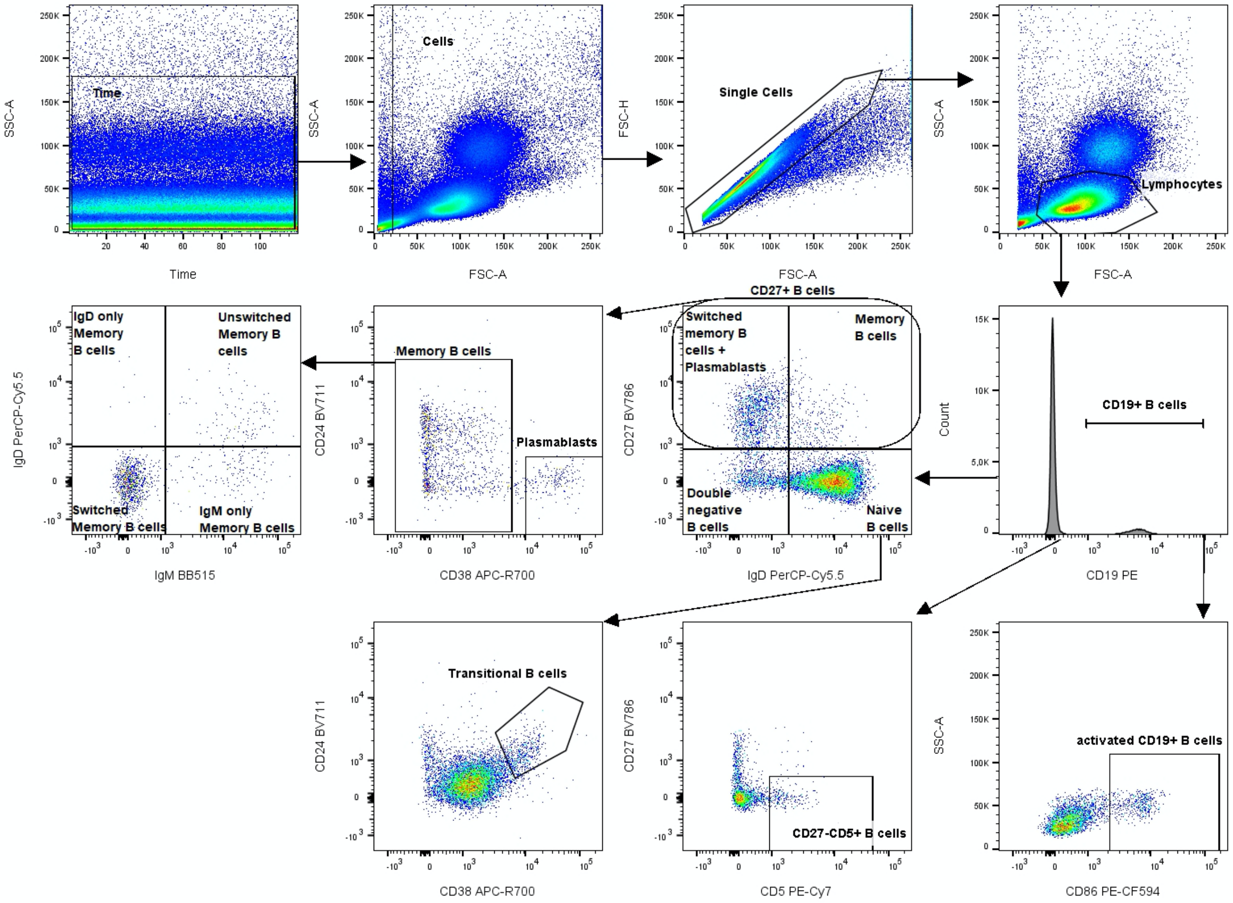

Supplementary Figure 1. Representative Plots for the gating strategy of B cell subsets. Lymphocytes were gated according to morphology (FSC-A vs. SSC-A). Beforehand, the timeline was checked for measurement artefacts and doublets were excluded. B cells were identified as $CD19^+$ in a histogram. Activated B cells were then identified in CD86 vs. SSC-A as $CD86^+$. $CD27^-CD5^+$ B cells were identified in CD5 vs. CD27. In IgD vs. CD27, naïve, memory, and double negative B cells were identified. In the IgD^-CD27^+ gate, switched and IgM-only memory B cells as well as plasmablasts are combined. For a further identification of memory B cells, both $CD27^+$ gates were combined and used to split memory B cells from plasmablasts (CD38 vs. CD24). These memory B cells were further divided into IgD-only, IgM-only, switched and unswitched memory B cells (IgM vs. IgD). Transitional B cells ($CD38^+CD24^+$) were gated from naïve B cells in a CD38 vs. CD24 plot.

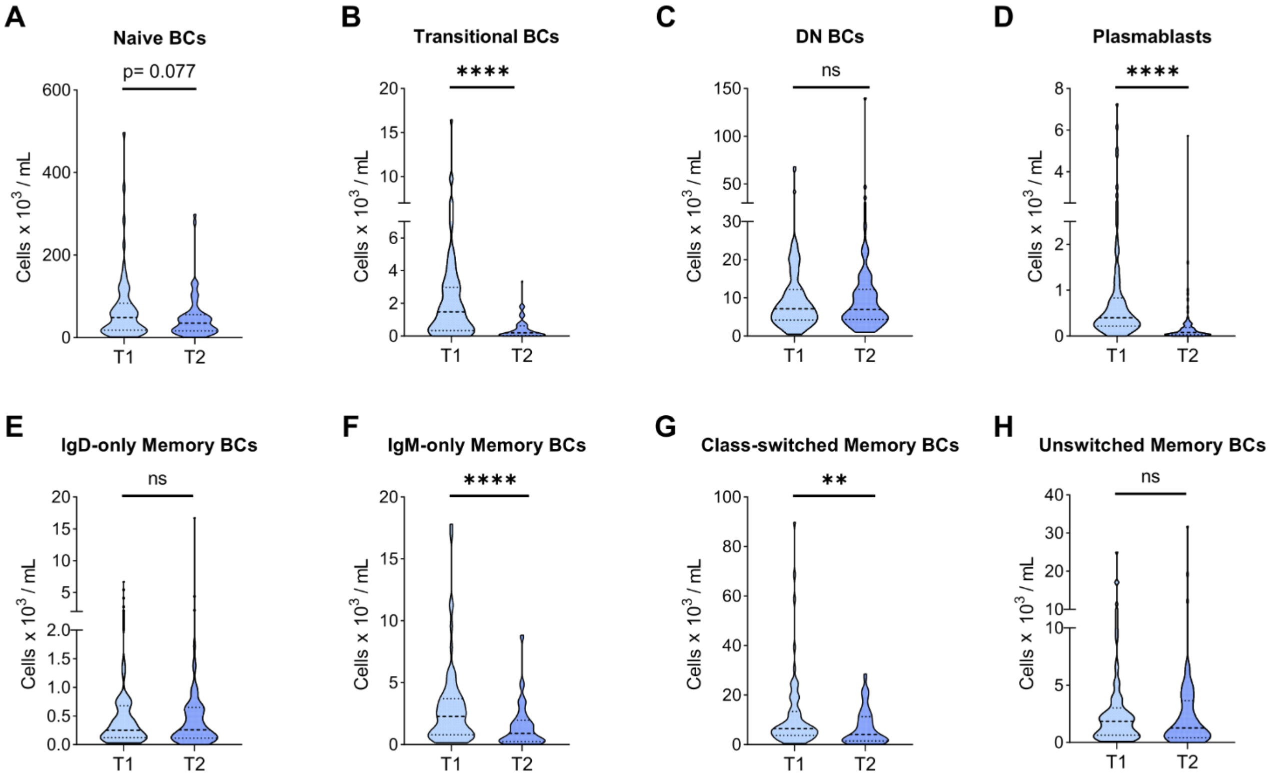

Supplementary Figure 2. Dynamics of BC subpopulation before and after one year of transplantation in absolute counts. Absolute numbers were calculated from total BC counts and relative frequencies of BC subpopulations for 71 KTRs before (T1) and one year after KT (T2). Violin plots show the distribution of counts of **(A)** naïve BCs, **(B)** transitional BCs, **(C)** double-negative BCs, **(D)** plasmablasts, **(E)** IgD-only memory BCs, **(F)** IgM-only memory BCs, **(G)** class-switched memory BCs, and **(H)** unswitched memory BCs per milliliter are given. Heavy dashed lines indicate the median, and light dashed lines indicate the interquartile range of data. Wilcoxon signed-rank test was used for calculating differences related to T1 (** $p< 0.01$; **** $p <0.0001$).

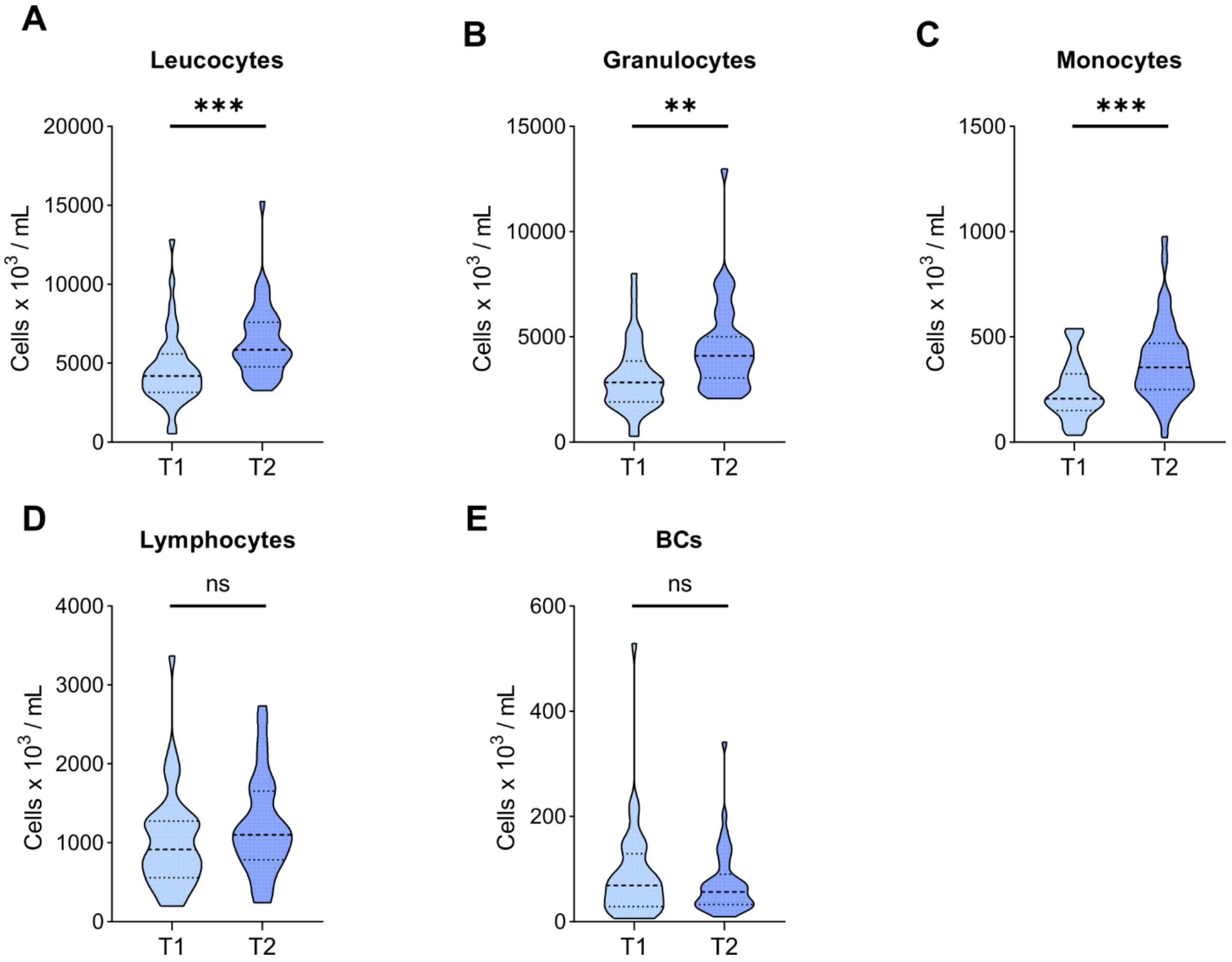

Supplementary Figure 3. Leucocytes and major leucocyte subpopulation counts in vaccination cohort. Whole blood of 40 patients was drawn before KT (T1) and one year after KT (T2), and stained for CD45. Major $CD45^+$ leucocyte subpopulations were differentiated according to forward and side scatter using flow cytometry and absolute numbers were obtained using 123count eBeads (Thermo Fisher Scientific). Absolute numbers of **(A)** leucocytes, **(B)** granulocytes, **(C)** monocytes, **(D)** lymphocytes, and **(E)** BCs are shown at T1 and T2. The plots shapes indicate the data distribution. Heavy and light dashed lines mark the median and interquartile range, respectively. Statistically significant differences are related to T1 and calculated using the Wilcoxon signed-rank test (** $p < 0.01$; *** $p < 0.001$).

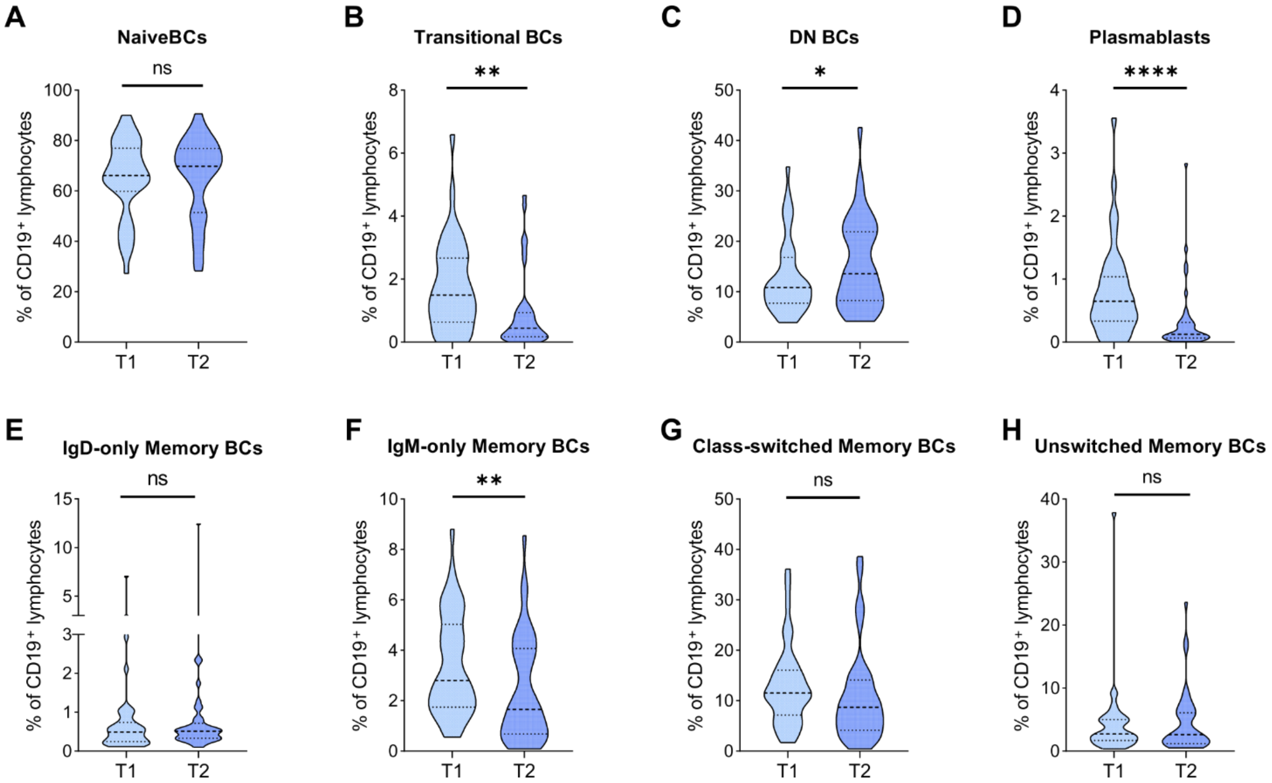

Supplementary Figure 4. Dynamics of BC subpopulation frequency before and one year transplantation in vaccination cohort. PBMCs were analyzed from 40 patients before (T1) and one year after KT (T2). Frequencies of **(A)** naïve BCs, **(B)** transitional BCs, **(C)** double-negative BCs, **(D)** plasmablasts, **(E)** IgD-only memory BCs, **(F)** IgM-only memory BCs, **(G)** class-switched memory BCs, and **(H)** unswitched memory BCs in $CD19^{+}$ lymphocytes are given as violin plots. Heavy dashed lines show the median and light dashed lines reflect the interquartile range. Wilcoxon signed-rank test was used for calculating differences related to T1 (* $p < 0.05$; ** $p < 0.01$; **** $p < 0.0001$).

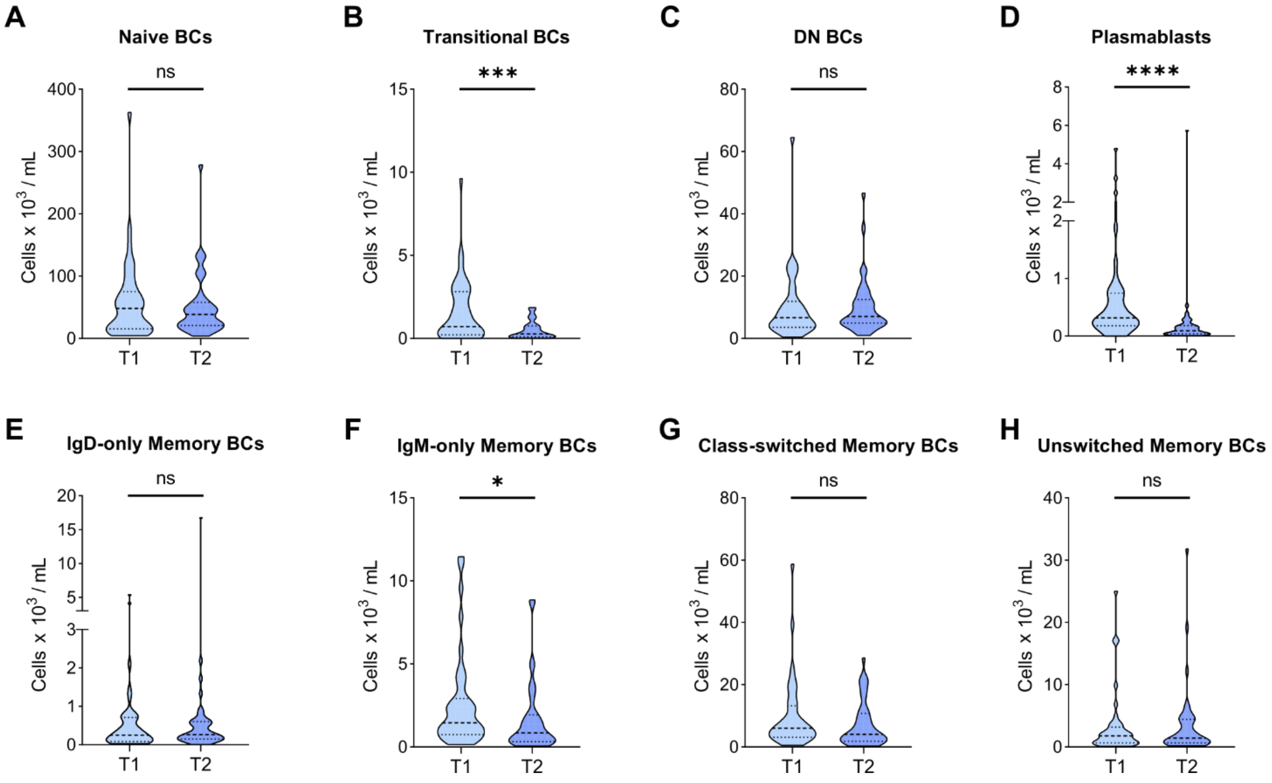

Supplementary Figure 5. Absolute numbers of BC subpopulation before and one year after transplantation in vaccination cohort. Absolute numbers were calculated from total BC counts and relative frequencies of BC subpopulations for 40 KTRs before (T1) and one year after KT (T2). Counts of **(A)** naïve BCs, **(B)** transitional BCs, **(C)** double-negative BCs, **(D)** plasmablasts, **(E)** IgD-only memory BCs, **(F)** IgM-only memory BCs, **(G)** class-switched memory BCs, and **(H)** unswitched memory BCs per milliliter are given. Data spread can be inferred from the plots shapes, and median and interquartile range are indicated by heavy and light dashed lines, respectively. Differences were calculated using Wilcoxon signed-rank test (* $p< 0.05$; *** $p< 0.001$; **** $p <0.0001$).

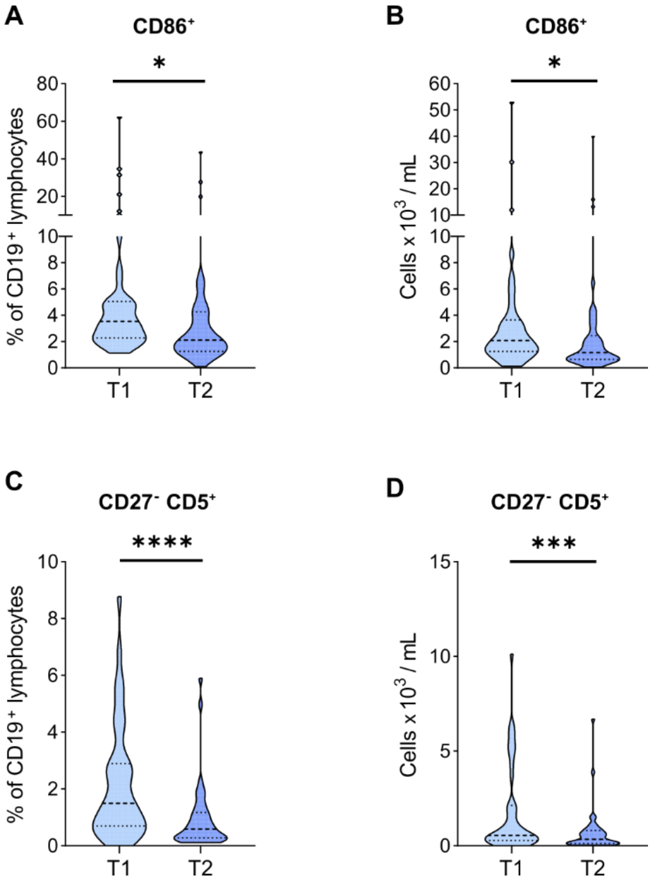

Supplementary Figure 6. Markers of activation and tolerance in BCs before and one year after KT in vaccination cohort. PBMCs of 40 KTRs were stained for CD86, CD27 and CD5 before (T1) and one year after KT (T2). Frequencies of **(A)** $CD86^+$ and **(C)** $CD27^-$ $CD5^+$ in $CD19^+$ lymphocytes are given. Absolute numbers per milliliter of **(B)** $CD86^+$ and **(D)** $CD27^-$ $CD5^+$ were calculated from their relative frequency and total BC counts. Heavy and light dashed lines specify medians and interquartile ranges, respectively. Differences between T1 and T2 were calculated using Wilcoxon-signed rank test (* p <0.05; *** p < 0.001; **** p <0.0001).
